# Probiotic Supplementation in the Neonatal Age Group and the Risk of Hospitalisation in the First Two Years: A Data Linkage Study from Western Australia

**DOI:** 10.3390/nu16132094

**Published:** 2024-06-30

**Authors:** Ravisha Srinivasjois, Amanuel Gebremedhin, Desiree Silva, Shripada C. Rao, Gizachew A. Tessema, Gavin Pereira

**Affiliations:** 1 Curtin School of Population Health, Curtin University, Perth, WA 6102, Australia; a.gebremedhin@curtin.edu.au (A.G.); gizachew.tessema@curtin.edu.au (G.A.T.); gavin.f.pereira@curtin.edu.au (G.P.); 2 Department of Paediatrics and Neonatology, Joondalup Health Campus, Perth, WA 6027, Australia; desiree.silva@telethonkids.org.au; 3 School of Medicine, University of Western Australia, Perth, WA 6005, Australia; shripada.rao@health.wa.gov.au; 4 EnAble Institute, Curtin University, Perth, WA 6102, Australia; 5 Neonatal Directorate, Child and Adolescent Health Service, Perth Children’s Hospital, Perth, WA 6009, Australia

**Keywords:** probiotic, neonate, infection, hospitalisation, gastrointestinal, respiratory

## Abstract

Background: Probiotic supplementation in preterm neonates is standard practice in many centres across the globe. The impact of probiotic supplementation in the neonatal age group on the risk of hospitalisation in infancy has not been reported previously. Methods: Infants born < 32 + 6 weeks of gestation in Western Australia were eligible for inclusion. We conducted a retrospective cohort study comparing data from before probiotic supplementation (Epoch 1: 1 December 2008–30 November 2010, n = 1238) versus after (Epoch 2: 1 June 2012–30 May 2014, n = 1422) on the risks of respiratory- and gastrointestinal infection–related hospitalisation. A subgroup analysis of infants born < 28 weeks of gestation was analysed separately for similar outcomes. Results: Compared to Epoch 1, an 8% reduction in incidence of hospitalisation up to 2 years after birth was observed in Epoch 2 (adjusted incidence rate ratio (IRR) of 0.92; 95% confidence interval (CI); 0.87–0.98), adjusted for gestational age, smoking, socioeconomic status, and maternal age. The rate of hospitalisation for infants born < 28 weeks of gestation was comparable in epochs 1 and 2. Conclusion: Infants exposed to probiotic supplementation in the neonatal period experience a reduced risk of hospitalisation in the first two years after discharge from the neonatal unit.

## 1. Introduction

The gut microbiome is a dynamic ecosystem that adjusts its composition depending on internal and external factors such as exposure to antibiotics, antacid medications, and diet. Any imbalance in gut microbiome, referred to as ‘dysbiosis’, can result in adverse health outcomes for the host [1]. Preterm infants are especially vulnerable to developing dysbiosis and related adverse outcomes [2,3]. Hence, probiotic supplementation has been trialled to attenuate gut dysbiosis and improve clinical outcomes.

Probiotics are live microorganisms that, when administered in adequate amounts, confer a health benefit on the host [4]. The colonised probiotic bacteria increase the secretory IgA, improve mucosal immunity, increase gut epithelial integrity, minimising bacterial translocation, and compete with the growth of pathogenic bacteria, thereby minimising their abundance in the gut [5]. The other beneficial pathways are through the production of mucin and short-chain fatty acids and through their effects on the immune function of the host [6]. The regular consumption of probiotics has been associated with the induction of regulatory T cells and the attenuation of nuclear transcription factor. Probiotics downregulate Toll-like receptor expression and inhibit tumour necrosis factor alfa (TNF-α), thus affecting the immune mediator pathways.

Many randomised controlled trials (RCTs) and meta-analyses have reported that probiotic supplementation in the neonatal period results in a significant reduction in the incidence of necrotizing enterocolitis (NEC), late onset sepsis, and feed intolerance in preterm infants [7,8,9,10,11]. Based on these findings, gut microbial modification using probiotic supplementation (PS) has now become a standard practice in many neonatal intensive care units [12]. The standard practice is to cease PS prior to hospital discharge. The length of time probiotic bacteria persist in the gut after the cessation of supplementation is not known. It is also unclear if the beneficial effects continue in infancy and early childhood even after the cessation of PS. Some studies have shown that enterally administered species of probiotic bacteria are identifiable in the stool until six months after the cessation of administration, but their abundance decreases over time [13]. The clinical implication of this observation is unclear. It is possible that adequate long-term colonisation is essential to the derivation of clinical benefits. It is also possible that the alterations to the immune system that occur during supplementation could result in long-term effects even after cessation. Altered immune function could result in increased/decreased risk of infections later in life, leading to excessive or decreased hospitalisations [6,14].

To address this issue, we carried out a retrospective cohort study with linkage analysis to answer the following question: Do preterm infants (patient) treated with probiotic supplements (intervention) in the neonatal age group experience changes in the risk of hospitalisation (outcome) in first two years of life compared to infants not exposed to probiotic supplementation (controls)?

## 2. Methods

Study design, setting, and population: This was a retrospectively conducted pre–post study comparing hospitalisation rates during the first two years of life among preterm infants born at less than 32 + 6 weeks of gestational age in Epoch 1 to Epoch 2. Epoch 1 (1 December 2008–30 November 2010) was the period when the infants did not receive PS. Epoch 2 (1 June 2012–30 May 2014) was the period when the infants received routine PS during the birth admission. These Epochs were chosen based on a publication by Patole et al., who had reported their short-term outcomes in the neonatal period [15]. The probiotic supplement used in the neonatal period was *Bifidobacterium Brevi* M-16 strain administered at a dose of 1.5–3 billion colony-forming units/day.

Source of clinical data: We used the Midwife Notification System (MNS) of the state of Western Australia (WA) to obtain birth-related information on the study infants. In WA, all live births and stillbirths from 20 weeks of gestation or with a birth weight of at least 400 g at public hospitals, private hospitals, or birth centres are included. Clinical details such as the gestational age, birth weight, and maternal complications such as gestational diabetes and preeclampsia are reported using the MNS.

We used the Hospital Morbidity Data System (HMDS) of WA to obtain relevant information on the health journey of infants born during the study period after discharge from birth-related hospitalisation. The HMDS uses the ICD-10 coding system to record the reasons for hospitalisation. We determined specific ICD- 10 codes for “any hospitalisation” and respiratory infection–related and GI infection–related hospitalisation. Details of the ICD codes used are presented in Appendix A.

**Figure 1:**
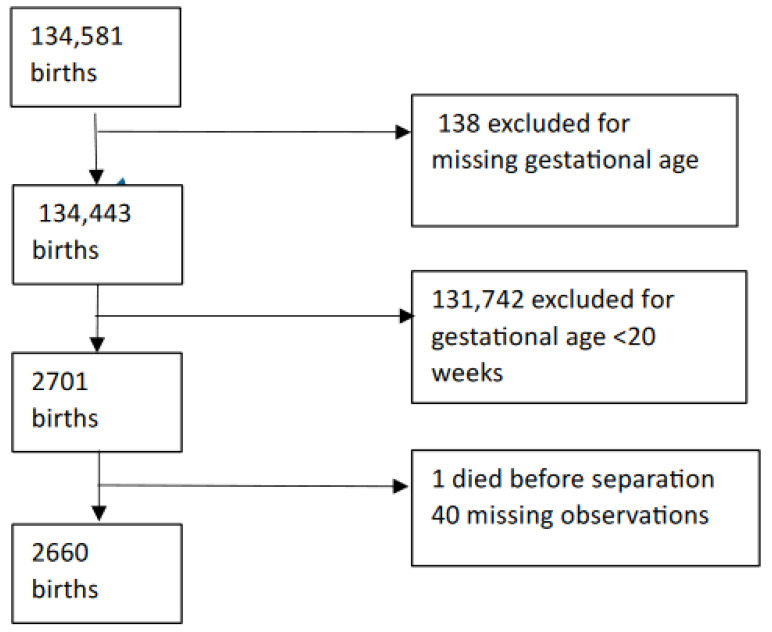
Selection log.

Exclusions: Records with missing gestational age and babies who died during neonatal hospitalisation were excluded from the analysis (Figure 1).

Outcome and exposure assessment: The primary outcome of interest was “any hospitalisation” during the first two years of life. Secondary outcomes were hospitalisations related to respiratory tract infections (RTI) or gastrointestinal infections. Exposure to probiotics was classified temporally by defining the two Epochs using complete dates of birth. Based on the results of systematic reviews that confirmed the benefits of probiotics [16] and a local pilot RCT that ensured the quality of the probiotic product [17], routine PS for preterm infants was commenced in 2012, and its short-term beneficial effects were reported recently [15]. The same cohort of infants (Epoch 1: no PS, Epoch 2: yes PS) was used for this study to evaluate if there are differences in their hospitalisation rates in the first two years of life.

Sensitivity analysis: A subgroup analysis was performed for the extremely preterm infants (born at < 28 weeks of gestation), given that they are the most vulnerable cohort of preterm infants.

Statistical analyses: For this analysis, we considered 30 June 2016 as the last date of hospitalisation, providing a duration of follow-up of two years.

To analyse the association between PS and the incidence of “any hospitalisation”, a negative binomial regression model was used with adjustment for gestational age (in weeks), maternal age (< 20, 20–24, 25–29, 30–34, 35–39, and > 40 years), ethnicity (Caucasian, Aboriginal, and other), smoking during pregnancy (yes or no), and socio-economic status. SES was derived by the Australian Bureau of Statistics as Socio Economic Indexes for Areas (SEIFA) at a geographical area for the maternal residence at the time of birth and categorised into quintiles (Australian Bureau of Statistics. Socio-Economic indexes for areas. Australian Bureau of statistics, 2013 http://www.abs.gov.au/websitedbs/censushome.nsf/home/seifa, accessed on 20 Nov 2023). The effect sizes were summarised as unadjusted incident rate ratios (IRR) and adjusted IRR (aIRR) with 95% confidence intervals (CI). The secondary outcomes (GI- and respiratory infection–related hospitalisations) were analysed using generalised linear models (GLM), Poisson family, with a logarithmic link function adjusting for similar variables as the main outcome.

Human research ethics approvals: Ethics approval for data linkage was obtained from Curtin University and the Department of Health, Western Australia Human research Committee, ref 2026/51.

## 3. Results

A total of 134,581 births were reported during the study periods. After exclusions, follow-up information was available on 1238 infants in Epoch 1 and 1422 in Epoch 2 and, hence, was used in the analysis (Appendix B).

The majority of infants were of Caucasian ethnicity (Table 1). The unadjusted incidence rate of “any hospitalisation” in Epoch 2 was smaller than in Epoch 1 (IRR 0.94, 95% CI 0.88–1.00). The incidence rate ratio was attenuated slightly after adjustment for gestational age, smoking, socioeconomic status, and maternal age (aIRR 0.92; 95% confidence interval (CI); 0.87–0.98) (Table 2). There was insufficient evidence for an association between PS and the incidence of any hospitalisation in the subgroup of infants born at < 28 weeks of gestation, but the sample size was small.

Risk of GI infection–related hospitalisation: The total number of GI infection–related hospitalisations were 40 and 50 in Epochs 1 and 2, respectively. The unadjusted and adjusted risks of GI infection–related hospitalisation in Epoch 2 were similar to those in Epoch 1 (Appendix C and Appendix D)

Risk of respiratory infection–related hospitalisation: The total number of respiratory-related hospitalisations were 278 and 252 in Epochs 1 and 2, respectively. The unadjusted risk of respiratory infection related hospitalisation in Epoch 2 was lower compared to Epoch 1. A reduction in respiratory-related hospitalisation was also observed after adjustment for GA, SES, maternal age, smoking, and ethnicity (aIRR 0.82 (95% CI 0.69: 0.98)) (Table 2). The results also indicated that a reduction in GA by one week increased the risk of hospitalisation due to respiratory cause by 6% (IRR 1.06 (95% CI 1.03, 1.09)).

## 4. Discussion

The results of this retrospective study with linkage analysis indicate that PS in very preterm infants during the neonatal period could reduce the incidence of “any hospitalisation” and “respiratory infection–related hospitalisation” in the first two years of life.

In addition to “any hospitalisation”, we explored hospitalisations specifically due to respiratory and gastrointestinal infections because they are the commonest causes of hospitalisations in infancy and early childhood [18], and probiotics have been shown to modulate secretary IgA, thereby improving respiratory and gut immunity [19,20]. Probiotics also have the potential to influence respiratory outcomes via the gut–lung axis [21], a form of immunological crosstalk between the gut microbiome and respiratory cells [22,23]. The recent Cochrane review that included 23 RCTs and a cluster RCT concluded that the regular administration of probiotics reduced the risk of upper respiratory infections in adults, children, and older people in the community, care facilities, schools, and hospitals [24]. Studies in neonatal mice have identified improved protection against respiratory syncytial virus after supplementation with a probiotic mixture consisting of *Lactobacilli* [25]. Qu et al. reported that the risk of bronchopulmonary dysplasia in preterm neonates was lower in infants exposed to probiotic supplementation in the neonatal period [26]. These benefits were noticeable while the patients were on probiotic supplementation. There is lack of information on whether such benefits can be sustained even after stopping the supplementation. The results of our study provide evidence supporting the hypothesis that the beneficial effects of probiotics may be sustained for up to two years after ceasing the supplementation.

Contrary to our expectations, PS was not associated with a decreased incidence of gastrointestinal infection-related outcomes. Our study also found no association between PS and hospitalisation rates in extremely preterm infants < 28 weeks of gestation (aIRR 0.93, CI: 0.85, 1.02). This could be due to the small sample size or a true finding. Further research with a larger sample size is needed to address these findings.

In WA, the most common cause of hospitalisation in the first 5 years of life was infection related. A report from Srinivasjois et al. identified that infection-related hospitalisation occurred in 8.9% of children in the first year and in 17.4% of children from 1 to 5 years of age [27]. Respiratory and gastrointestinal aetiology were the common reasons for hospitalisation. Gestational age was inversely related to the risk of hospitalisation.

Similar to the previous studies, the incidence of hospitalisations due to any cause or respiratory/gastrointestinal infection–related hospitalisation was higher in non-Caucasian ethnicity [28,29,30]. Future studies should specifically evaluate the efficacy and safety of probiotic supplementation in this high-risk population [31].

Evidence is accumulating that gut microbiota influence the structure and function of the brain and other systems through action on the gut–brain axis, gut–lung axis, and gut–liver axis [21,32,33,34,35,36,37,38]. However, meta-analysis of RCTs has shown that PS in the neonatal period does not translate into improved growth or neurodevelopmental outcomes for preterm infants [39]. One possible reason for such a lack of benefit could be the cessation of probiotics prior to discharge from neonatal units.

To address the issue scientifically, recently completed and currently ongoing placebo controlled RCTs in preterm infants [8,40] should evaluate infection related hospitalisations in early childhood. If they confirm benefits, that would indicate the long-term effects of probiotics even after cessation. Future RCTs in preterm infants should compare the continuation of probiotic supplementation versus ceasing intervention prior to discharge from neonatal units. If better outcomes are observed in the “continuation group”, that would provide support to the argument that to achieve sustained benefits, probiotic supplementation needs to be continued even after discharge from neonatal units.

A recent systematic review [13] identified four RCTs (n = 605 infants) that evaluated whether oral probiotic supplementation in the neonatal period results in sustained gut colonisation with probiotic bacteria at or beyond 6 months after its cessation. Of them, three RCTs (n = 471) showed the presence of intestinal probiotic bacteria at 6–12 months. They concluded that the low certainty of evidence suggests that probiotic supplementation in the neonatal period may result in sustained gut colonisation 6–12 months post-cessation, but not at 24 months. Neonatal units that routinely use probiotics should endeavour to analyse the gut microbiota of such infants until 1–2 years of age to know if colonisation persists long after the cessation of supplementation and correlates with clinical outcomes.

To our knowledge, this is the first study to report the sustained effects of PS beyond the neonatal age group even after ceasing supplementation. The data is extensive, state-wide, and accurate. Data linkage in Western Australia has been shown to be effective and reliable [41]. We think that it is unlikely that the state experienced a downward trend for hospitalisations in Epoch 2 compared to Epoch 1, although our methodology does not allow for investigating that possibility. Hence, we believe that the data presented here are a true reflection of the real outcomes experienced by infants born at different gestational ages.

To the best of our knowledge, the correlation between gut microbiome modification in the neonatal period and later risk of hospitalisation has not been reported previously. The limitations of the study include a lack of clinical information on the use of over-the-counter probiotic supplementation in infants after discharge from the neonatal unit. We believe that the likelihood of ex-preterm infants being exposed to over-the-counter probiotics is low. We do not have information on the number of patients on supplemental formula and diets containing probiotic bacteria. Unconfirmed errors and systematic errors in coding for the main causes of hospitalisation could not be excluded, although they are considered unlikely.

## 5. Conclusions

The results of this study provide evidence of a reduction in the risk of any hospitalisation (and, specifically, a reduction in respiratory-related hospitalisation) in the first two years of life post-cessation of PS with *Bifidobacterium breve* M-16 in preterm infants. There was no effect of PS on the risk of gastrointestinal-related hospitalisation.

Additional well-designed studies with due consideration of the risk factors that affect the development of the gut microbiome are required to further understand the longer-term effects of PS initiated in the neonatal period.

## Figures and Tables

**Table 1:** Demographic characteristics.

Variable	N (%)	Epoch 1 1238 (%)	Epoch 2 1422 (%)	*p* Value ^1^
Maternal age (years)				0.003
25–29	689 (26)	309 (25)	380 (27)	
<20	208 (7.8)	120 (9.7)	88 (6.2)	
20–24	350 (13)	172 (14)	178 (13)	
30–34	919 (35)	405 (33)	514 (36)	
35–39	418 (16)	204 (16)	214 (15)	
>40	76 (2.9)	28 (2.3)	48 (3.4)	
Ethnicity				
Caucasian	1814 (68)	895 (72)	919 (65)	
Others	846 (32)	343 (28)	503 (35)	
Smoking during pregnancy				
Yes No	510 (19)	235 (19)	275 (19)	0.800
	2150 (81)	1003 (81)	1147 (81)	
Gestational age (weeks)				0.049
<24	535 (20)	261 (21)	274 (19)	
24–28	555 (21)	277 (22)	278 (20)	
28–32	1570 (59)	600 (57)	870 (61)	
SES percentiles ^2^				
<20	460 (17)	212 (17)	248 (17)	
20–39	555 (21)	255 (21)	300 (21)	
40–59	526 (20)	250 (20)	276 (19)	
60–79	583 (22)	260 (21)	323 (23)	
>80	536 (20)	261 (21)	275 (19)	

^1^ Pearson’s chi-squared test. ^2^ Socio-economic status.

**Table 2:** Risk of hospitalisation.

	Hospitalisation (Unadjusted)	Adjusted for GA, Smoking, Ethnicity, Maternal Age, and SES	GI Infection (Unadjusted)	GI Infection (Adjusted for Smoking, Ethnicity, Maternal Age and SES)	Respiratory Infection (Unadjusted)	Respiratory Infection (Adjusted for Smoking, Ethnicity, Maternal Age, and SES)
	IRR ^1^ (95% CI ^2^)	IRR (95% CI)				
Epoch 1						
Epoch 2	0.94 (0.88, 1.00)	0.92 (0.87, 0.98)	1.10 (0.73, 1.68)	1.00 (0.66, 1.52)	0.80 (0.67, 0.94)	0.82 (0.69, 0.98)

^1^—Incidence rate ratio, ^2^—Confidence interval.

## Data Availability

Data are not available publicly but may be available to a third party after appropriate ethics approval through the Western Australia Department of Health Human Research Ethics.

## Appendix A

**Table A1:** List of ICD-10 codes used for hospitalisation and GI infection–related hospitalisation.

Respiratory Infection Related Hospitalisations						
**J10–18**	J10	**J20–22**	J20	**J06.9**	**J22**	**J98.8**
	J10.0		J20.0			
	J10.1		J20.1			
	J10.8		J20.2			
	J11		J20.3			
	J11.0		J20.4			
	J11.1		J20.5			
	J11.8		J20.6			
	J12		J20.7			
	J12.0		J20.8			
	J12.1		J20.9			
	J12.2		J21			
	J12.3		J21.0			
	J12.8		J21.8			
	J12.9		J21.9			
	J13		J22			
	J14					
	J15					
	J15.0					
	J15.1					
	J15.2					
	J15.3					
	J15.4					
	J15.5					
	J15.6					
	J15.7					
	J15.8					
	J15.9					
	J16					
	J16.0					
	J16.8					
	J17					
	J17.0					
	J17.1					
	J17.2					
	J17.3					
	J17.8					
	J18					

**GI infection–related hospitalisation.**

A08
A08.0
A08.1
A08.11
A08.19
A08.2
A08.3
A08.31
A08.32
A08.39
A08.4
A08.8
A09

## Appendix B

**Table A2:** Selection log.

Variable	N	Excluded (n)	Epoch 1 (n)	Epoch 2 (n)
	134,581		62,552	72,029
GA missing		138	62,490	71,953
GA < 22 or > 32 weeks	134,443		1267	1434
2-year follow-up	2701	131,742	1266	1434
Records of death before separation	2700	1	1	0
Other exclusions	2699	9	27	12
Numbers included			1238	1422

## Appendix C

**Table A3:** Risk of hospitalisation under various statistical models.

	Model 1	Adjusted for GA	Adjusted for GA, Smoking, and Ethnicity	Adjusted for GA, Smoking, Ethnicity, Maternal Age, and SES	Restricted for GA ≤ 28 Weeks	Restricted to GA ≤ 28 Weeks, and Adjusted for GA
Characteristic	IRR ^1^ (95% CI ^2^)	IRR (95% CI)	IRR (95% CI)	IRR (95% CI)	IRR (95% CI)	IRR (95% CI)
Epoch 1						
Epoch 2	0.94 (0.88, 1.00)	0.93 (0.87, 0.99)	0.92 (0.87, 0.98)	0.92 (0.87, 0.98)	0.96 (0.87, 1.05)	0.93 (0.85, 1.02)
GA (reversed)		0.97 (0.96, 0.97)	0.97 (0.96, 0.97)	0.97 (0.96, 0.97)		0.88 (0.87, 0.90)
Smoking						
No						
Yes			1.09 (1.00, 1.18)	1.07 (0.99, 1.17)		
<20				-		
20–39				0.90 (0.82, 1.00)		
40–59				0.96 (0.87, 1.07)		
60–79				0.87 (0.78, 0.96)		
>80				0.95 (0.86, 1.06)		
Maternal age (years)						
25–29				ref		
<20				0.91 (0.80, 1.03)		
20–24				0.96 (0.86, 1.06)		
30–34				0.89 (0.82, 0.96)		
35–39				0.87 (0.78, 0.96)		
40+				0.83 (0.68, 1.02)		

^1^ Incidence rate ratio, ^2^ Confidence interval.

## Appendix D

**Table A4:** Sensitivity analysis. Risk of GI- and respiratory-related hospitalisation.

	GI Infection (Unadjusted)	GI Infection (Adjusted for Smoking, Ethnicity, Maternal Age, and SES)	Respiratory Infection (Unadjusted)	Respiratory Infection (Adjusted for Smoking, Ethnicity, Maternal Age, and SES)
Epoch 1				
Epoch 2	1.10 (0.73, 1.68)	1.00 (0.66, 1.52)	0.80 (0.67, 0.94)	0.82 (0.69, 0.98)
GA reversed		0.88 (0.82, 0.93)		0.94 (0.91, 0.97)
Smoking				
No				
Yes		1.51 (0.91, 2.49)		1.21 (0.98, 1.50)
SES quintiles				
<20				
20–39		0.64 (0.32, 1.21)		0.86 (0.66, 1.12)
40–59		0.96 (0.51, 1.78)		0.86 (0.66, 1.12)
60–79		0.76 (0.40, 1.43)		0.74 (0.56, 0.98)
>80		0.62 (0.29, 1.27)		0.82 (0.62, 1.09)
Maternal age (yrs)				
25–29				
<20		0.47 (0.19, 1.01)		0.91 (0.66, 1.23)
20–24		0.65 (0.22, 1.21)		1.15 (0.89, 1.49)
30–34		0.66 (0.39, 1.10)		0.78 (0.62, 0.98)
35–39		0.39 (0.16, 0.84)		0.69 (0.50, 0.92)
>40+		0.61 (0.10, 2.02)		0.51 (0.23, 0.96)
